# The New Field of Network Physiology: Building the Human Physiolome

**DOI:** 10.3389/fnetp.2021.711778

**Published:** 2021-06-30

**Authors:** Plamen Ch. Ivanov

**Affiliations:** ^1^ Keck Laboratory for Network Physiology, Department of Physics, Boston University, Boston, MA, United States; ^2^ Harvard Medical School and Division of Sleep Medicine, Brigham and Women’s Hospital, Boston, MA, United States; ^3^ Bulgarian Academy of Sciences, Institute of Solid State Physics, Sofia, Bulgaria

**Keywords:** network physiology, dynamic networks, complex systems, control, AI, sensory networks, big data, human physiolome

## State-of-the-Art and Fundamental Questions

The human organism comprises various physiological and organ systems, each with its own structural organization and functional complexity, leading to complex, transient, fluctuating and nonlinear output dynamics (Ivanov et al., 1996, 1999a; Goldberger et al., 2002). Basic physiology and clinical medicine widely employ a reductionist approach, and consider health and disease through the prism of the structural organization and dynamics of individual organ systems. Further, physiological states and functions at the organism level are traditionally defined by the dynamics of organ systems, their modulation and changes in response to transitions in biochemical signaling and neuro-autonomic regulation due to internal, external and pathologic perturbations (Amaral et al., 1998; Ivanov et al., 1999b; Bunde et al., 2000; Kantelhardt et al., 2002; Karasik et al., 2002; Hu et al., 2004b; Ivanov et al., 2004; Schmitt et al., 2009; Schumann et al., 2010).

However, the human organism is an integrated network, where multi-component physiological systems, each with its own regulatory mechanism, continuously interact to coordinate their functions. Coordinated network interactions among organs are essential to generating distinct physiological states and maintaining health. Physiological interactions occur at multiple levels of integration and across spatiotemporal scales to optimize organ functions and synchronize their dynamics at the organism level. Often manifested as synchronized bursting activities with certain time delays, these interactions are mediated by various signaling pathways that work in parallel to facilitate stochastic and nonlinear feedbacks (Ivanov et al., 1998; Hausdorff et al., 2001) across scales leading to different coupling forms (Bartsch et al., 2014; Bartsch and Ivanov, 2014). Having structurally intact and functioning systems is not sufficient to maintain health.

In addition to the state of individual organ systems, coordinated network interactions among systems and sub-systems are essential to generate distinct physiologic states and behaviors at the organism level, such as wake, sleep and sleep stages, rest and exercise, stress and anxiety, cognition, consciousness and unconsciousness. Disrupting organ communications can lead to dysfunction of individual systems or trigger a cascade of failures leading to a breakdown and collapse of the entire organism, as observed under clinical conditions such as sepsis, coma and multiple organ failure (Buchman, 2006; Moorman et al., 2016; Shashikumar et al., 2017; Foreman et al., 2021). Yet, despite the vast progress and achievements in systems biology and integrative physiology in the last decades, and the importance to basic physiology and clinical practice, we do not know the principles and mechanisms through which diverse systems and sub-systems in the human body dynamically interact as a network and integrate their functions to generate physiological states in health and disease.

The new multi-disciplinary field of Network Physiology aims to address these fundamental questions (Bashan et al., 2012; Ivanov and Bartsch, 2014). In addition to defining health and disease through structural, dynamical and regulatory changes in individual physiological systems, the new conceptual framework of Network Physiology focuses on the coordination and network interactions among diverse organ systems and sub-systems as a hallmark of physiologic state and function.

A fundamental problem in physical, biological, and physiological systems is to understand phenomena where global behaviors across systems emerge out of networked interactions among dynamically changing entities with coupling forms that are often nonlinear and change as a function of time. Early attempts to study multiple sub-systems within the cardiovascular system (Guyton et al., 1972), later extended to other systems (Coleman and Randall, 1983), were modeled on electric circuit diagrams that simply sum up individual measurements from separate physiologic experiments and could not begin to account for the transient dynamics and emergent non-linear behaviors which are hallmarks of human physiology. Efforts in recent years to understand specific physiological interactions such as cardio-respiratory coupling (Bartsch et al., 2012; Angelone and Coulter, 1964; Rosenblum et al., 1996; Schäfer et al., 1998; Mrowka et al., 2000; Pikovsky et al., 2001; Song and Lehrer, 2003; Garcia-Retortillo et al., 2019) (e.g., we all know our hearts race when we breathe in) did not address the collective behavior of organ-to-organ interactions.

**FIGURE 1:**
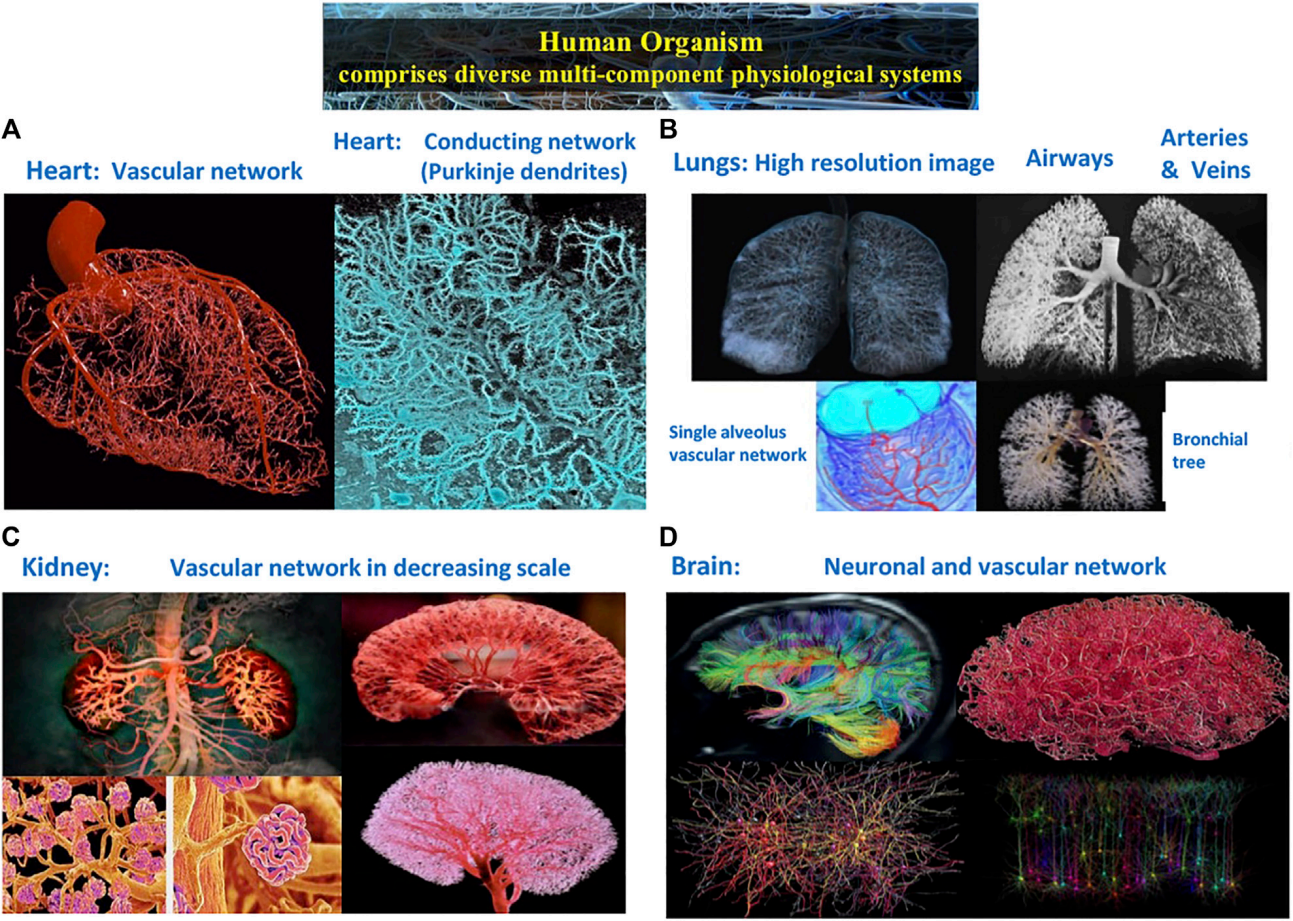
Complex structural and functional networks underlie the dynamics and mechanisms of regulation across spatial and temporal scales in multi-component physiological and organ systems. **(A)** Heart: vascular network and conducting network of Purkinje dendrites embedded in the myocardial muscle. **(B)** Lungs: airways and vascular networks from the bronchial tree to a single alveolus. **(C)** Kidney: vascular network revealing cortex structure at large scales, nephrons at intermediate scales and single glomeruli at small scales. **(D)** Brain: diffusion tensor image of connectivity micro-structure networks showing the location, orientation, and anisotropy of white matter tracts; vascular networks; and neuronal population networks representing levels of activation for individual neural cells from real time electron microscope imaging.

**FIGURE 2:**
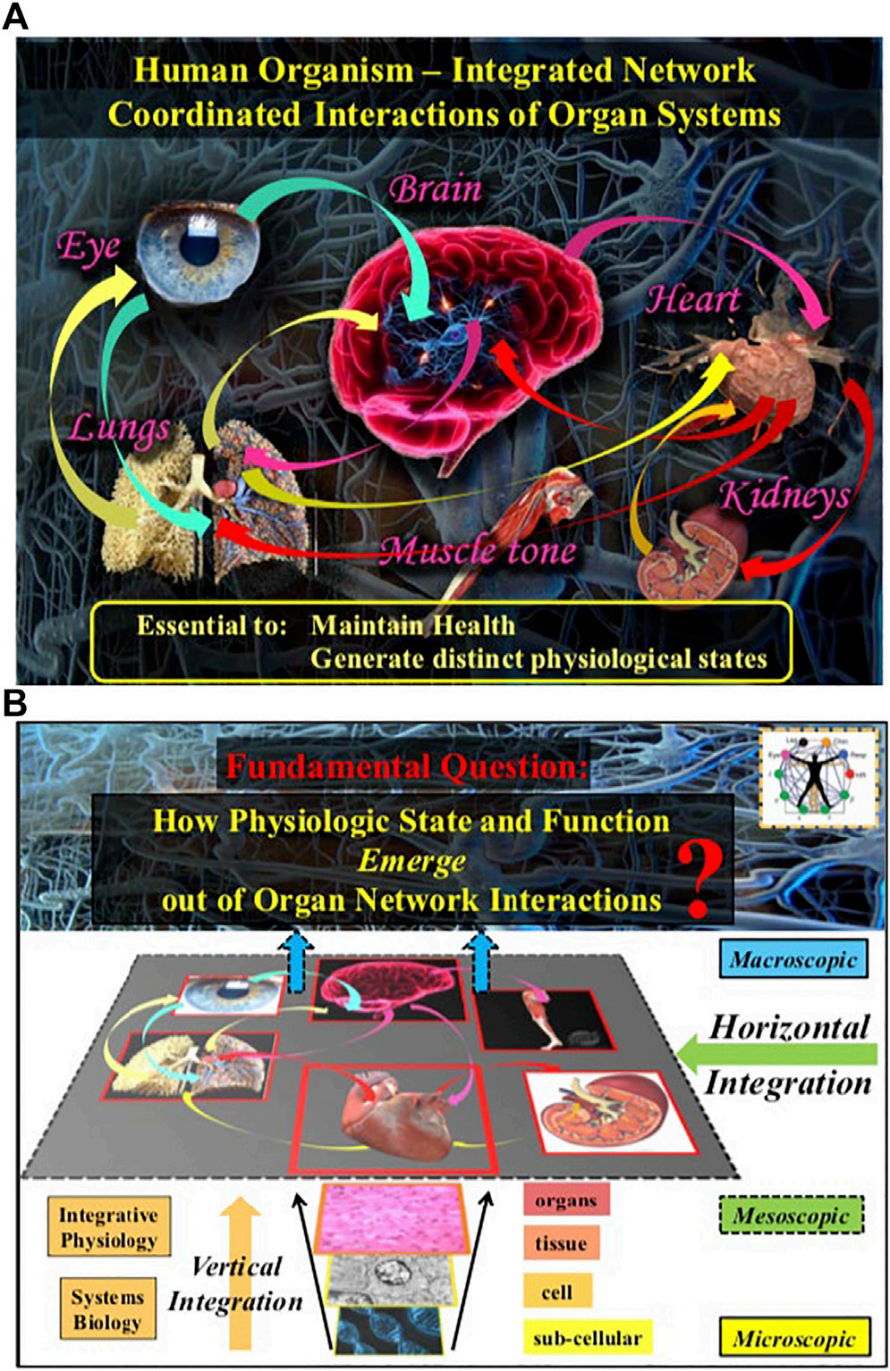
The human organism is an integrated network where diverse physiological and organ systems continuously interact to optimize and coordinate their functions. **(A)** Network interactions across spatial levels and temporal scales within systems and among systems are essential to generate various physiological states and to maintain health. **(B)** A fundamental question in Network Physiology is how physiological states and functions emerge out of vertical and horizontal network integration from the sub-cellular to the organism level.

Identifying and quantifying these interactions is a major challenge due to the complex dynamics of organ systems (Ivanov et al., 2001; Suki et al., 2003; Hu et al., 2004a). Such complexity arises from intrinsic interactions of multi-component cellular and neuronal sub-systems that build and regulate each organ in the human body (Figure 1), leading to intermittent, scale-invariant and nonlinear output signals. This is further compounded by various coupling and feedback interactions between organ systems that continuously vary in time (Stankovski et al., 2015), the nature of which is not understood. In fact, it was recently discovered that two organ systems can communicate through several forms of coupling that simultaneously coexist (Bartsch et al., 2014; Bartsch and Ivanov, 2014). This poses a barrier to our understanding of how organs integrate their functions to generate emergent behavior of the human body as a single entity able to adapt to internal and external perturbations, and mantain homeostasis (Fossion et al., 2018). The framework of computational neuroscience and systems physiology, which focus on pathways of neuron-to-neuron signaling and on integration within organ systems, do not provide adequate tools and are not of help here (Lehnertz et al., 2020). Even the most recent advances in systems biology and integrative physiology (Large, 2011) continue to focus on how genetic/cellular interactions relate to function at the single tissue and organ level; occasionally, physiologists will leap directly from micro-level sub-cellular and cellular insights on genomic, proteomic and metabolic interactions to “macroscopic” epidemiological observations. There is a wide gap in research efforts and knowledge at the “mesoscopic” level of horizontal network interactions across organ systems and sub-systems essential to maintaining health (Figure 2). The new field of Network Physiology has emerged to fill this gap, and to address the fundamental question of how physiological systems synchronize and integrate their dynamics as a network to optimize functions and to maintain health.

Two major stumbling blocks hamper investigations in this direction: 1) long-term, continuous, parallel recordings from multiple organ systems are not readily available across different physiologic states and conditions (Finazzi et al., 2018); collecting such data in both ambulatory and clinical ICU/hospital environment is particularly problematic because medical devices are often not interoperable; 2) there are no well-established analytic methodology, computational tools, theoretical framework capable of probing organ interactions from continuous streams of data, that are simultaneously applicable to diverse organ systems with different output dynamics. The complex, multi-scale dynamics of organ systems make it extremely challenging to identify and quantify the network of organ interactions.

In dynamic networks of physiological interactions links represent coordination and synchronization between systems and sub-systems, and exhibit transient characteristics. A key question is how physiological states and functions emerge out of the collective network dynamics of integrated systems. While network structure may play a role in generating various states and functions, different global behaviors at the organism level can emerge from the same network topology due to changes in systems dynamics (network nodes) and modulations in the functional form of physiologic interactions (network links). This poses new challenges in developing generalized methodology adequate to quantify complex dynamics of networks where nodes represent systems with diverse dynamics, interacting through different forms of coupling that continuously change in time with transitions across states and conditions. Novel concepts and approaches derived from recent advances in network theory, coupled dynamical systems, statistical and computational physics, biomedical informatics, signal processing and biological engineering show promise to provide new insights into the complexity of physiological structure and function in health and disease, bridging across levels of integration sub-cellular signaling with inter-cellular interactions and communications among integrated organ systems and sub-systems. These advances form first building blocks in the methodological formalism and theoretical framework necessary to address the problems and challenges in the field of Network Physiology.

Network Physiology focuses on inferring coupling and dynamical interactions among organ systems based on continuous streams of synchronous recordings of key physiologic parameters and output signals from multiple systems. In contrast to traditional complex network theory, where edges/links are constant and represent static graphs of association, novel approaches in Network Physiology have to take into consideration 1) the complex dynamics of individual systems (network nodes), 2) dynamical aspects of network links representing organ communications in real time, 3) on the evolution of organ interactions with time and 4) emergence of collective network behavior in response to changes in physiologic states and conditions. This new field will integrate empirical and theoretical knowledge across disciplines with the aim to understand in different contexts, from extensive data analysis and modeling approaches to clinical practice, how diverse organs, physiological systems and sub-systems dynamically interact as a network from the cellular to the organism level to produce various physiological states and functions in health and disease.

**FIGURE 3:**
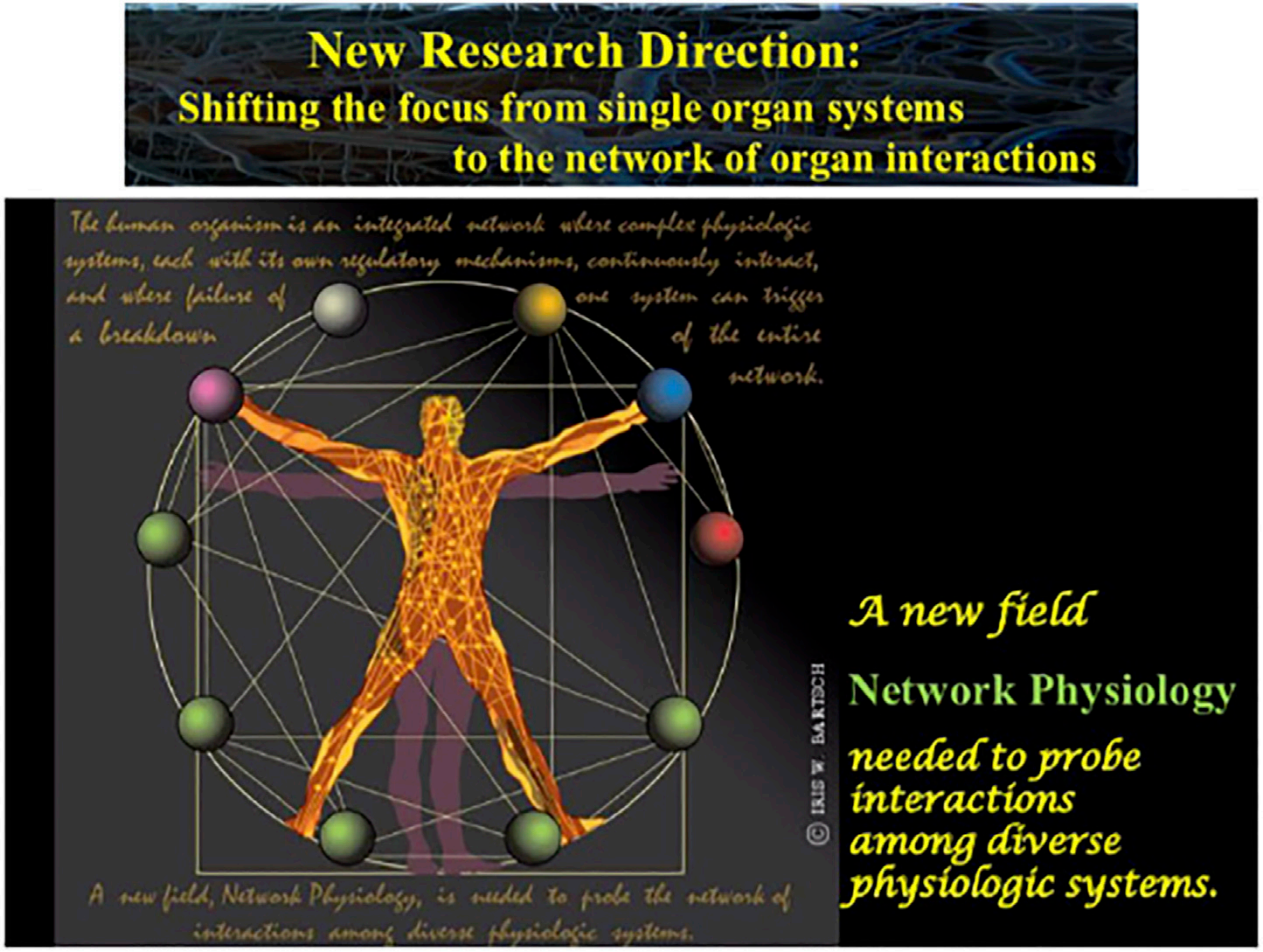
A new field, Network Physiology, has emerged, shifting the focus from single organ systems to the network of physiologic interactions with the aim to uncover basic laws of communication and principles of integration in networks of diverse physiological systems and their role in generating global behaviors at the organism level.

In classical graph theory, network nodes and network links are static and represent statistical correlations and dependence rather than dynamical coupling. Dynamical aspects in classical network theory arise from removing/adding links or nodes and from diffusion processes of flow on a fixed network, where emphasis is given on the consequences of network topology and structure on networks function to transmit information. In contrast, in Network Physiology, links represent dynamical coupling and coordination between diverse systems and sub-systems and have transient characteristics. Changes in the dynamicsof physiological systems (network nodes) can propagate via ‘elastic’ time-varying links to affect the dynamics of other nodes, and thus, alter the behavior of the entire network. A fundamental question is how to quantify, predict and control emergent global behaviors in temporal multiplex networks of diverse dynamic systems interacting simultaneously through various functional forms of coupling. In such adaptive networks, markedly different global behaviors can emerge from the same network topology due to minor temporal changes in the dynamics of a node or in the functional form of a link (Ivanov and Bartsch, 2014). This directly relates to the question of how a variety of physiologic states and functions emerge out of the collective dynamics of integrated physiological and organ systems (Bartsch et al., 2015; Liu et al., 2015b; Rizzo et al., 2020; Ivanov et al., 2017, 2021b). This poses new challenges to further develop generalized methodology adequate to quantify complex dynamics of networks where nodes are not identical but represent diverse dynamical systems with diverse forms of coupling which continuously change in time. Such investigations are not simply an application of established concepts and approaches in complex networks theory to existing fields of biomedical research. Because of the new type of problems, the specificity of related challenges, and the necessity of new theoretical framework and interdisciplinary efforts, Network Physiology has developed into a new field of research (Figure 3).

The scope of Network Physiology extends far beyond applying knowledge from one field (statistical physics, applied mathematics, informatics, network theory) to solve problems in another (systems biology, neuroscience, physiology and medicine). New computational and analytical approaches are needed to extract information from complex data, to infer transient interactions between dynamically changing systems, and to quantify global behavior at the organism level generated by networks of interactions that are function of time. In fact, in recent years, we have already witnessed the broad impact of introducing novel concepts and methods derived from modern statistical physics and network theory to biology and medicine, shifting the paradigm from reductionism to a new integrative framework essential to address fundamentally new problems in systems biology (Yao et al., 2019; Prats-Puig et al., 2020; Corkey and Deeney, 2020; Rizi et al., 2021; Barajas-Martínez et al., 2020), neuroscience (Castelluzzo et al., 2020; Pa¨eske et al., 2020; Fesce, 2020; Stramaglia et al., 2021), physiology (Podobnik et al., 2020; Zmazek et al., 2021), clinical medicine (Loscalzo and Barabasi, 2011; Delussi et al., 2020; Li et al., 2020; Liu et al., 2020; McNorgan et al., 2020; Tan et al., 2020; Haug et al., 2021; Liu et al., 2021) and even drug discovery (Hopkins, 2008). A central focus of research within this integrative framework is the interplay between structural connectivity and functional dependency, a key problem in neuroscience, brain research (Bullmore and Sporns, 2009; Gallos et al., 2012; Rothkegel and Lehnertz, 2014; Liu et al., 2015a; Bolton et al., 2020; Wang and Liu, 2020) and human physiology (Pereira-Ferrero et al., 2019; Lavanga et al., 2020; Barajas-Martínez et al., 2021; Gao et al., 2018; Balagué et al., 2020; Porta et al., 2017; Lioi et al., 2017; Jiang et al., 2021). As a result, new physical models have been motivated and proposed to investigate the dynamical consequences of adaptive networks (de Arcangelis et al., 2006; Millman et al., 2010; Gómez-Gardenes et al., 2011; Komarov and Pikovsky, 2013; Pecora et al., 2014; Zhang et al., 2015; Wellman et al., 2018; Wang et al., 2019; Lombardi et al., 2020; Masamura et al., 2020; Suki et al., 2020; Rowland Adams and Stefanovska, 2021; Polizzi et al., 2021), which in turn trigger more theoretical questions. These synergetic effects certainly establish Network Physiology as a new field in the landscape of contemporary biomedical and interdisciplinary research. Understanding the relationship, conceptual difference, the broad horizon and impact of Network Physiology is important to facilitate an active and productive dialog among physicists, biologists, physiologists, neuroscientists and medical clinicians.

The field of Network Physiology will draw on and facilitate the development of multiple areas of empirical and theoretical, basic and clinical research—from advanced methods for nonlinear dynamics and synchronization phenomena, theory of dynamical systems and adaptive networks, data-driven models of complex systems and their interactions, control theory in dynamic networks, information theory for coupling inference and causality for non-stationary and non-linear systems, new generation of data-intensive AI and machine learning algorithms for inference of network dynamics and function, biomedical engineering of sensors networks and human-machine interfaces—to numerous areas in basic physiology and clinical medicine, including proteomic and metabolic networks, networks of cell assembles, neuronal populations, networks of the autonomic and peripheral nervous systems, brain structural and functional networks, biomechanical networks in tissues, networks in the cardio-vascular and respiratory systems, network structures and dynamics in the kidneys and renal system, networks of skeletal muscle groups and muscle fibers, pairwise and network interactions of organ systems and sub-systems, and their manifestations in aging, exercise and sports, as well as in numerous clinical and pathological conditions with impact on multiple physiological systems in the human body, such as concussion and traumatic brain injury, cardiac arrest, sleep and neurodegenerative disorders, diabetes and obesity, maternal-fetal and neonatal care, sepsis, coma and multiple organ failure.

**FIGURE 4:**
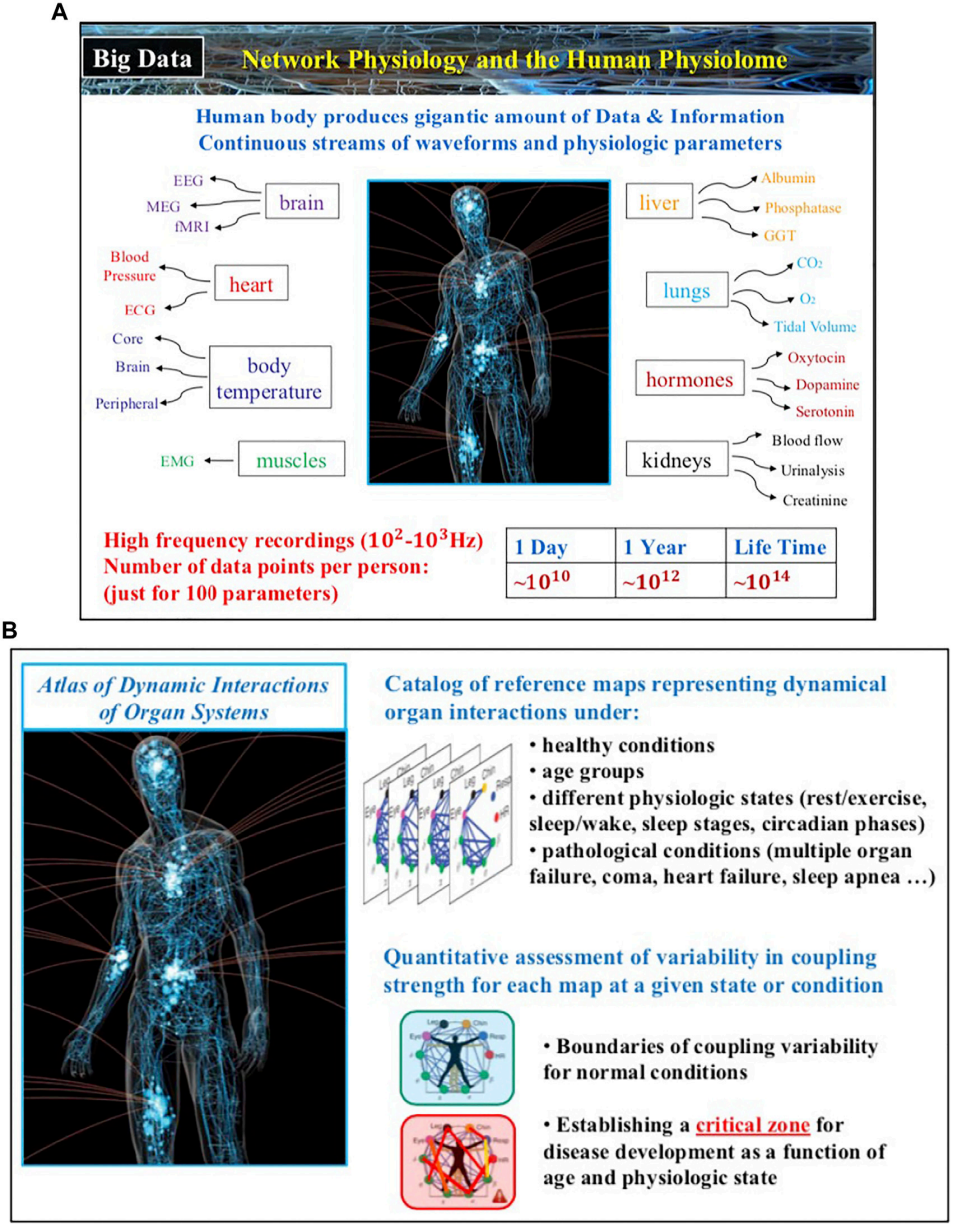
The human body generates continuous streams of physiological signals as output dynamics of various systems and physiological parameters that contain a wealth of information about the state of individual systems and the nature of their network interactions. **(A)** In 24 h just one hundred basic physiologic parameters recorded with 100 Hz generate 10^9^–10^10^ data points, of the same order as the number of nucleotides in the human genome. **(B)** Novel methods and approaches within the framework of Network Physiology aim to establish associations between distinct physiologic states and pathological conditions with the structure and dynamics in physiological networks, and thus, lay the foundations of the Human Physiolome, a first of a kind Big Data of blueprint reference network maps representing states and conditions through network interactions across levels in the human body.

The field of Network Physiology also involves bioengineering research and development of novel biomedical device platforms for synchronized high-frequency recordings from multiple physiological systems both in the clinical ICU and hospital environment as well as networks of wearable sensors for continuous measurement of physiological parameters in free ambulatory conditions. Integrated networks of clinical monitoring devices and wearable sensors that provide high-precision, synchronous signals are essential to establish causality and pathways of dynamical interactions in networks of physiological systems, to track the evolution of these interactions across states and conditions, to develop new class of network-based markers for diagnosis and prognosis of clinical conditions and critical events. Thus, future developments in Network Physiology will lead to establishing a new kind of Big Data, the Human Physiolome (Figure 4), containing large-scale signals from multiple systems and an associated blueprint repository of hundreds of network maps representing physiological systems interactions for different states, conditions and diseases. New machine learning and AI algorithms trained to identify both topological characteristics as well as temporal dynamics of physiological networks, able to predict hierarchical re-organization and cascades of breakdown in dynamic networks, have to be developed to classify states, functions and conditions based on network physiology maps from large populations of subjects.

## Major Challenges

Quantifying networks of physiological interactions poses major challenges. These challenges arise from several levels of complexity inherent to the dynamics of organ systems. Physiological systems exhibit non-stationary, intermittent, scale-invariant and nonlinear behaviors (Ivanov et al., 1999a; Ivanov et al., 2001). Their output dynamics transiently change in time with different physiologic states and under pathologic conditions in response to changes in the underlying control mechanisms. The structural and neural control networks that underlie each physiologic organ system include many individual components, connected through nonlinear interactions, that lead to high degree of freedom. This complexity is further compounded by various coupling and feedback interactions among different systems, the nature of which is not understood.

Moreover, physiological systems operate on a broad range of time scales from milliseconds to hours and exhibit different types of output dynamics—oscillatory, stochastic or mixed—and thus, earlier concepts of treating them as coupled chaotic oscillators need to be extended (Ashkenazy et al., 2002; Chen et al., 2006; Xu et al., 2006). Further, each integrated physiological system exhibits multiple simultaneous interactions and different forms of coupling with other systems, where interactions among systems vary in time (Bartsch and Ivanov, 2014; Bartsch et al., 2012; Bartsch et al., 2014). This leads to a transient multi-layer network structure consisting of distinct physiologic networks. Importantly, global network dynamics of the entire organismcannot be simply expressed as a sum of the behaviors of individual systems, and can be strongly influenced by minor changes in the behavior of one system and/or in the relative strength of interactions with other systems, even when network topology remains unchanged.

There are several fundamental and challenging questions in the field of Network Physiology. Physiological systems at the sub-cellular, tissue and organism level exhibit bursting dynamics (Ferrari et al., 2015) that result from molecular and cellular level signaling processes. A key question is whether synchronous bursts in systems dynamics can reveal fundamental information regarding the nature of network communications among systems. Specifically, what is the role of synchronized bursting dynamics in mediating neural control and flow of information between physiologically relevant brain rhythms and corresponding neuronal populations within and across brain areas, between cortical rhythms and other physiological systems, and among key organ systems. Can studies of transient synchronous bursts in systems dynamics reveal fundamental laws of network communications among systems. Can we uncover dynamical characteristics of brain- organ and organ-organ networks as a new signature of physiologic control, and can we establish robust associations of network structure and dynamics with physiologic states and functions at the organism level. Are there basic universal principles of integration in networks of diverse physiological systems that underly interactions between motifs, modules, sub-networks and networks formed by physiological systems at different levels and time scales. How physiological states and functions emerge from network interactions among diverse systems, what degree of coupling, link intensity distribution and coordination between systems dynamics is necessary to facilitate a physiological state at the organism level. How physiological networks hierarchically re-organize with transitions from one physiological state to another in response to changes in autonomic regulation, and what is the “critical zone” of variability in network interactions beyond which critical events occur leading to cascades of breakdown across the organism.

These fundamental questions pose challenges to developing new methodology and theoretical framework to identify and quantify dynamical interactions among systems with very different characteristics and signal outputs. There is an urgent need for adopting a cross-scale perspective to investigate the multi-scale regulatory mechanisms underlying the overall network physiology and its relation to physiological states and functions, and to address the heterogeneity, multi-modality and complexity of physiological processes. We need rigorous mathematical and algorithmic techniques that can extract causal interdependencies between systems across different scales while overcoming various noise sources. Progress in this direction will require new strategies to quantify time-varying information flow among diverse physiological processes across scales, and determine how it influences the global dynamics of complex physiological networks. Intrinsically related to future efforts on quantifying causal dependencies and control principles in biological and physiological networks, it will be essential to develop robust optimization algorithms capable to reconstruct or infer the structure and dynamics of complex interdependent networks while overcoming partial observability, noise induced defects and adversarial interventions caused by external perturbations, bacterial or viral infections.

Recent research efforts have focused on temporal networks (Holme and Saramäki, 2012), where traditional graph approaches to static network topology are extended to time-dependent structures, and are employed to investigate new phenomena related to changes in fundamental properties of networks, including the loss of transitivity and the emergence of time ordering of links (Holme and Saramäki, 2012). However, the inherent complexity of physiological systems and the problems that arise from network physiology are beyond the scope of the current-state-of-the-art in temporal networks. Specifically, current approaches to temporal networks do not account for the complex dynamics of individual physiological systems (network nodes) and for the heterogeneity of physiological networks comprised of diverse systems where coupling forms (individual network links) vary in time. Moreover, the current formalism employed in temporal networks requires a well-defined time-scale, which is not adequate for physiologic networks where scale-invariant dynamics and temporal feedbacks over a broad range of time scales are well-known hallmarks of integrated physiological systems. Currently, there is no established analytic instrumentarium and theoretical framework suitable to probe networks comprised of diverse systems with different output dynamics, operating on different time scales, and to quantify dynamic networks of organ interactions from continuous streams of noisy and transient signals.

Further, despite the increased need for smart healthcare sensing systems that monitor patients’ body balance, currently there is no coherent theory that facilitates the modeling of human physiological processes and the design and optimization of future healthcare cyber-physical systems (Bogdan and Marculescu, 2011; Xue and Bogdan, 2017; Bogdan, 2019). Utilizing new generation machine learning and AI algorithms, healthcare cyber-physical systems are expected to measure and mine the patient’s physiological state based on available continuous sensing, quantify risk indices corresponding to the onset of abnormality, signal the need for critical medical intervention in real-time by communicating patient’s medical information via a network from an individual to the hospital, and most importantly control (actuate) a network of vital health signals (e.g., cardiac pacing, insulin level, blood pressure) within personalized homeostasis.

It is also important to note current limitations, when one explores uncharted territory through the perspectives of Network Physiology. The progress towards a reliable network-based approach to disease is still limited by the incompleteness of the available data on protein–protein interactions, metabolic networks, information of biological regulatory pathways and organ interactions that are heavily relying on large scale biomedical experiments and streams of continuous physiological signals (Barabási et al., 2011; Cohen et al., 2021). Meanwhile, as research moves towards the dynamic interactome (Przytycka et al., 2010), it would certainly require new advances in temporal and adaptive networks to probe temporal variations in network topology and function. Network Physiology is still at an early stage (network building phase), where broad-scale empirical investigations are needed to establish a general framework to identify and define dynamical links among physiological systems, and to construct the specific physiological networks that dictate particular integrative functions. Since physiological systems communicate via complex mechanisms manifested through various functional forms of coupling, there is a need to integrate distinct forms of pair-wise physiologic interactions into a general framework that unites approaches from nonlinear dynamics, information theory and machine learning. Empirical investigations in Network Physiology will foster new development of data-driven modeling and theoretical approaches to provide mechanistic insights and elucidate principles of nonlinear control in physiological networks. This in turn will stimulate the development of new data-science methodology with broad impact on both basic biomedical research and clinical practice.

## Current Progress

To address these challenges, recent nonlinear methods based on phase synchronization (Schäfer et al., 1998; Song et al., 1998; Pikovsky et al., 2001; Chen et al., 2006; Bartsch et al., 2012; Bartsch and Ivanov, 2014), coherence (Chorlian et al., 2009; McCraty et al., 2009; Bian et al., 2014; Kralemann et al., 2014; Piper et al., 2014; Kerkman et al., 2020), complex wavelets (La Rocca et al., 2021), mutual information (Faes et al., 2014; Valente et al., 2018; Antonacci et al., 2020), transfer entropy (Dumont et al., 2004; Faes et al., 2015; Valenza et al., 2016a; Valenza et al., 2016b; Lucchini et al., 2020) and Granger causality (Stramaglia et al., 2014) have been proposed to infer nonlinear interactions between pairs of dynamical systems. Efforts have focused on extending these methods to quantify direct or indirect interactions, the strength and directionality of links and the functional forms of coupling in physiological networks. A novel concept of time delay stability was introduced to extract information from synchronous bursting activity across systems, and to identify and quantify transient physiologic interactions among diverse organ systems with distinct output dynamics (Bashan et al., 2012; Liu et al., 2015b). In recent years, multi-disciplinary research efforts have made significant contributions that led to discoveries with potential for broad clinical applications, including: novel applications of complex networks theory to ask fundamentally new questions in systems biology; human disease and co-morbidity networks; new physics of synchronization phenomena in networks of oscillators; new insights in neural networks and brain structural and functional connectivity; innovative methods to probe complexity in physiological time series of individual systems and the impact of individual systems on the dynamics of the entire physiologic network; dynamical networks of organ systems and functional forms of coupling; and clinical applications derived from networks of physiological interactions.

Novel computational tools and analytic formalism recently developed in the field of Network Physiology have added new dimensions to our understanding of physiologic states and functions. The network physiology perspective has redefined physiologic states from point of view of dynamic networks of organ interactions. Utilizing this new perspective, recent studies have focused on 1) investigating brain-brain network interactions across distinct brain rhythms and locations, and their relation to new aspects of neural plasticity in response to changes in physiologic state; 2) characterizing dynamical features of brain-organ communications as a new signature of neuro-autonomic control; 3) establishing basic principles underlying coordinated organ-organ communications, and 4) constructing first dynamic maps of physiological systems and organ interactions across distinct physiologic states (Bashan et al., 2012; Bartsch et al., 2012; Bartsch and Ivanov, 2014; Ivanov and Bartsch, 2014; Liu et al., 2015a; Liu et al., 2015b; Bartsch et al., 2015; Lin et al., 2016; Ivanov et al., 2017, 2021b; Dvir et al., 2018; dos Santos Lima et al., 2019; Lin et al., 2020; Rizzo et al., 2020; Ivanov et al., 2021a; Balagué et al., 2020). Pioneering investigations have made first insights into structural and functional connectivity of physiologic networks underlying individual organ systems and their sub-systems (Tass et al., 1998; Bullmore and Sporns, 2009; Gallos et al., 2012; Liu et al., 2015a; Neufang and Akhrif, 2020; Cohen et al., 2021; Cook et al., 2021), and how global behaviors at the organism level, different physiologic states and functions arise out of networked interactions among organ systems to generate health or disease (Bashan et al., 2012; Ivanov and Bartsch, 2014; Karavaev et al., 2020; Pernice et al., 2020; Tecchio et al., 2020; Wood et al., 2020; Zavala et al., 2020; Angelova et al., 2021; Guillet et al., 2021; Morales et al., 2021; Mukli et al., 2021). This has led to identifying first associations of distinct physiologic states and conditions with specific network topology and temporal characteristics of organ interactions (Bashan et al., 2012; Ivanov and Bartsch, 2014). It was discovered that brain-organ interactions have preferred channels of communication (frequency bands) that are specific for each organ (Bartsch et al., 2015). Recent studies that focused on networks of brain–heart interactions identified new aspects of coupling and neuro-autonomic feedback mechanisms (Valenza et al., 2016b; Lin et al., 2016). By developing the theoretical framework necessary to uncover basic principles of 1) integration among diverse physiologic systems that leads to complex physiologic functions at the organism level, and of 2) hierarchical reorganization of physiological networks and their evolution across states and conditions, investigations in the field of Network Physiology provide first building blocks of an atlas of dynamic interactions among physiological systems in the human body and lay the foundation of the Human Physiolome.

## Impacts and Future Development

The unique fundamental questions we address in Network Physiology will change the current paradigm of defining physiologic states, health and disease by shifting the focus from single organs to the network of physiologic interactions. Investigations in the field will help unravel the mystery of how health emerges as a result of network interactions among systems. Coordinated interdisciplinary research efforts in the field will establish basic principles of organ integration essential to generate emergent behaviors at the organism level, and to facilitate responses and adaptation to internal and external perturbations, and thus, will redefine physiological states and functions in health and disease through unique network maps of physiologic interactions.

Novel mathematical and computational methods will be developed to address the complexity of physiological systems, to facilitate empirical findings of physiological interactions, and to build the first theoretical framework for investigations of emerging global behaviors in networks of dynamical systems. This will directly impact areas of applied math, computer and data science, and network theory as 1) we develop new techniques for physiological data analyses, and 2) introduce new generation network models of dynamical systems with time-dependent interactions to uncover mechanisms of hierarchical integration, global network evolution across states and re-organization between distinct network modules, motifs, and communities of integrated physiological systems and sub-systems.

Future developments in Network Physiology will revolutionize our knowledge and understanding of the mechanisms that regulate and coordinate organ-to-organ interactions; establish first quantitative measures of the interactions between diverse organ systems and of their collective network behavior; uncover relations between physiologic states and patterns of organ network interactions; establish the hierarchical structure of physiological networks, the mechanism of network control and re-organization with states, conditions and disease; and thereby open entirely new areas of research at the interface of computational and data science, applied mathematics and physics, AI and bioengineering, physiology and medicine.

**FIGURE 5:**
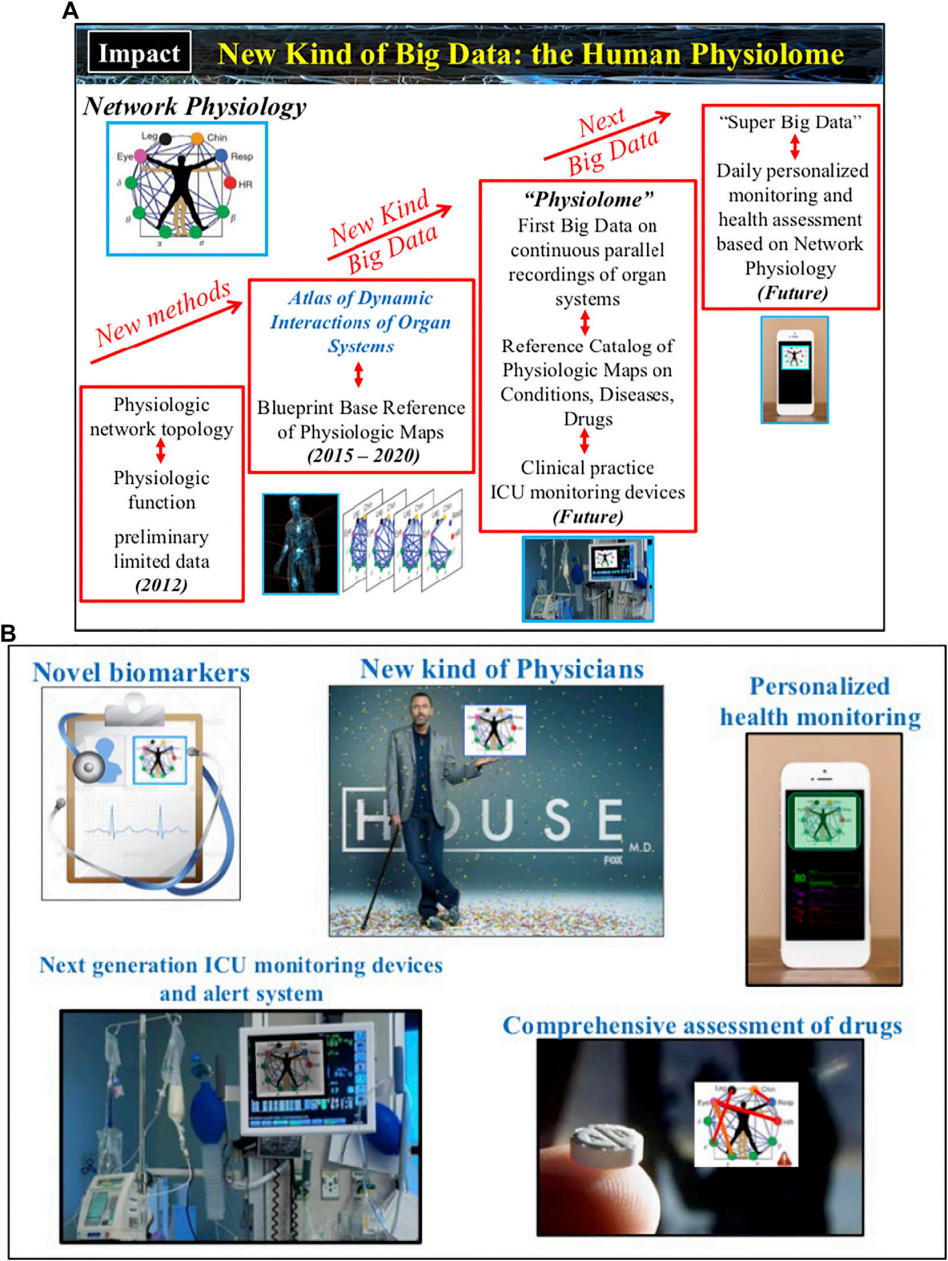
Developments in Network Physiology will revolutionize our knowledge and understanding of the principles underlying systems’ communications and their integration as a network, and the mechanisms that coordinate and control organ-to-organ interactions. **(A)** Current technological advances and findings of association between physiologic networks structure and dynamics with physiological function open the horizon to develop a new kind of Big Data and build the Human Physiolome — a dynamic atlas of network maps representing physiologic interactions across levels and systems in the human body under health and disease. **(B)** A broad range of applications will follow: novel network-based biomarkers and taxonomy of disease; next generation integrated biomedical devices and sensor networks to facilitate prediction of critical events and guide treatment strategies; comprehensive assessment of drugs effects not only on individual systems but also on the interactions among systems; personalized health monitoring; new educational and training tools for physicians and clinicians.

A number of potential basic science and medical innovations could follow (Figure 5): Novel methods tailored to infer coupling, causality and directionality of interactions among nonlinear systems with time-varying dynamics; New generation of AI and machine learning algorithms trained to simultaneously respond to both spatial and temporal features of dynamic networks; New class of data-driven network models to study mechanisms of emergent global network behaviors and phase transitions; Novel biomarkers based on organ network interactions for early diagnosis, age-related risk assessment and pathological conditions; Next-generation ICU monitoring/alert systems that incorporates maps of organ network interactions and AI algorithms to track real-time changes of states and conditions; New, comprehensive ways to assess the effects of medical treatment strategies and drugs, not just on a targeted organ system but on the coupling between organs; New network-based taxonomy of disease and co-morbidity networks; New kind of Big Data, the Human Physiolome (Figure 4), consisting of continuous long-term synchronous recordings from multiple physiological systems and the corresponding blueprint reference network maps (representing physiologic interactions across temporal and spatial scales from the sub-cellular to the organism level) that are associated with basic physiological states (wake and sleep, sleep stages, rest and exercise, stress and anxiety, cognition etc.), conditions (age groups, gender, race etc.), and disease (neurodegenerative and metabolic disorders, sleep and circadian disorders, cancer, diabetes and obesity, concussion and brain trauma, comma, cardiac arrest, sepsis, multiple organ failure etc.); New level of real-time personalized health monitoring; Establish the mathematical foundation and theoretical framework of the new interdisciplinary field of Network Physiology. Further, the uncovered laws of communication between organ systems, basic principles of integration in physiological networks and mechanisms of network control that lead to emergence of global network behaviors at the organism level will open new avenues of research and applications in the fields of bio-engineering, electronics and robotics, where next generation intelligent electronic and robotic systems, as well as swarms of bots, will implement algorithms derived from principles of interactions and network organization among physiological systems to execute versatile and complex tasks.

## Multi-Disciplinary Community and a New Journal

The fundamental questions and challenges in the field of Network Physiology have drawn attention and have generated interest in a diverse community of research scientists across a broad range of disciplines and fields from applied mathematics, physics, data science and biomedical engineering to neuroscience, physiology and clinical medicine. Several collections of articles with focus on Network Physiology published in leading interdisciplinary journals, including New Journal of Physics (Ivanov, 2016), Physiological Measurement (Ivanov, 2017) and Frontiers in Physiology (Ivanov et al., 2019), have facilitated nucleation of ideas, identifying key problems, exchange of concepts and methodology, and helped outline new frontiers of synergetic research in the field. Conferences hosted by the International Summer Institute on Network Physiology at the Lake Como School for Advanced Studies (ISINP, 2017; ISINP, 2019) have served as a forum to present basic research and clinical studies, discuss challenges and future developments, train the next generation of young scientists, and foster collaborations among groups and institutions across countries, thus, establishing a world community working in this newly emerging field.

In April 2021, Frontiers, a leading open access publisher and open science platform, has launched Frontiers in Network Physiology, the first journal publishing rigorously peer-reviewed research and dedicated to furthering our understanding of network physiology. This multidisciplinary, open-access journal is at the forefront of communicating impactful scientific discoveries to academics and clinicians. The journal provides a platform for articles covering a range of physiological systems from the metabolic, sub-cellular and cellular level to integrated organ systems and the entire organism, and will publish cutting-edge empirical and theoretical works, discuss the challenges, current frontiers and future developments in the field of Network Physiology.

Frontiers in Network Physiology welcomes both basic research and clinical studies, and aims to promote data-driven discoveries of laws and control mechanisms that underlie physiologic network interactions under both health and pathological conditions. Of particular interest will be new approaches to identify and quantify forms of physiologic coupling as well as developing new and little-explored areas of network science of relevance to integrated physiological systems. The journal will also foster the development of research on next generation network-based diagnostic/prognostic markers and treatment strategies, as well as the development of new integrated biomedical engineering device platforms and sensory networks for multi-systems data recording and analysis. The scope of the journal encompasses a broad range of topics, including: Functional forms of physiologic coupling, time variation and effects of pair-wise interactions on the dynamics and control of individual systems; Network studies on structural and dynamical aspects of physiological sub-systems and systems that transcend space and time scales; Information flow on network topology in relation to cellular and neuronal assemblies and autonomic control of organ systems; Networks comprised of diverse physiological systems and associations between physiologic network structure and physiologic function; Basic principles of hierarchical network organization; Evolution of pair-wise coupling and network topology with transitions across physiologic states; Role of time-dependent network interactions for emergent transitions in network topology and function; Networks of physiological networks transcending interactions of sub-systems to interactions among organs; Manipulation, control and global dynamics of networks in response to clinical treatment; Cascades of failure across systems as encountered in critical care; Development of physiologically inspired AI algorithms, electronic and robotic systems based on the laws and principles of physiologic network interactions.

The conceptual framework of Network Physiology and the integrative approaches this new field offers to explore emerging cooperative phenomena and critical states in networks of diverse dynamical systems with nonlinear and time-varying interactions open new exciting horizons in both basic and applied sciences with broad impact on data science, biomedical technology and clinical practice. Advances in Network Physiology will revolutionize our understanding of health and disease. We invite the community to join this new multi-disciplinary science, and take part in an exciting journey of new discoveries and applications to build the Human Physiolome.
